# Posterior Reversible Encephalopathy Syndrome in Pediatric Cancer: Clinical and Radiologic Findings

**DOI:** 10.1200/JGO.17.00089

**Published:** 2017-12-04

**Authors:** Saadiya Javed Khan, Arjumand Ali Arshad, Mohammad Bilal Fayyaz, Islah ud din Mirza

**Affiliations:** All authors: Shaukat Khanum Cancer Hospital and Research Center, Lahore, Pakistan.

## Abstract

**Purpose:**

Posterior reversible encephalopathy syndrome (PRES) is associated with a range of medical conditions and medications. In this retrospective analysis, we present 19 pediatric patients with PRES who had undergone chemotherapy.

**Methods:**

We identified four female and 15 male patients diagnosed with PRES on the basis of clinical and radiologic features. Patient charts were reviewed from January 2013 to June 2016 after authorization from the institutional review board.

**Results:**

The average age of patients with PRES was 7 years. Primary diagnoses were non-Hodgkin lymphoma (n = 9), acute pre–B-cell leukemia (n = 5), relapsed pre–B-cell leukemia (n = 2), Hodgkin lymphoma (n = 2), and Ewing sarcoma (n = 1). PRES occurred during induction chemotherapy in 12 patients. Sixteen patients had hypertension when they developed PRES. Most of these patients (n = 13) were receiving corticosteroids on diagnosis of PRES. Common clinical features were hypertension, seizures, and altered mental status. With the exclusion of three patients, all others required antiepileptic therapy. Ten of these patients underwent additional magnetic resonance imaging. Ten patients are still alive.

**Conclusion:**

In patients who presented to our center with signs and symptoms of hypertension, seizures, visual loss, or altered mental status, PRES was mostly seen in those who were undergoing systemic and intrathecal chemotherapy. Approximately 40% of the patients had reversal of clinical and radiologic findings. Antiepileptic medications were discontinued after being seizure free for approximately 6 months.

## INTRODUCTION

In 1996, Hinchey et al^1^ described a syndrome of acute, but reversible clinical features that included headaches, mental status change, seizures, hypertension, and acute visual disturbance associated with radiologic changes on magnetic resonance imaging (MRI). This clinicoradiologic disease pattern was termed posterior leukoencephalopathy syndrome. Typical temporary changes in subcortical white matter seen on T2-weighted and fluid-attenuated inversion recovery (FLAIR) images are characteristic for this syndrome, which is now better known as posterior reversible encephalopathy syndrome (PRES).^2,3^ PRES has been involved with a variety of medical conditions, including cancers, eclampsia, solid organ transplants, renal diseases, and autoimmune disorders.^1,4^ It is also seen in pediatric patients with cancer.^5-8^ This single-institution retrospective study analyzed a cohort of pediatric patients with cancer and PRES to describe the clinicoradiologic features and outcomes of children with cancer who develop PRES during treatment.

## METHODS

We identified 19 patients diagnosed with PRES at our institution during January 2013 to June 2016. PRES was defined as the presence of at least one classical clinical symptom, such as hypertension, visual disturbance, altered mental status, seizure, and cortical blindness, in combination with MRI abnormalities.^9^ A radiologist reviewed the MRI findings of all patients included in this study. Medical history, clinical characteristics, chemotherapy schedules, diagnosis of PRES, management, and clinical outcome data were collected retrospectively from the hospital medical records after institutional review board approval.

## RESULTS

The study cohort included 15 male and four female patients. The mean age at the time of PRES onset was 7 years (median, 5 years; range, 2.5 to 16 years). Details of patient characteristics are listed in Tables 1 and 2. Primary diagnosis of these patients included non–Hodgkin lymphoma (n = 9), acute pre–B-cell leukemia (n = 5), relapsed pre–B-cell leukemia (n = 2), Hodgkin lymphoma (n = 2), and Ewing sarcoma (n = 1). None of the patients had CNS primary malignancy or involvement. In this study, one patient with Ewing sarcoma was not treated with either corticosteroids or intrathecal chemotherapy. PRES occurred during induction chemotherapy in 12 patients.

**Table 1:**
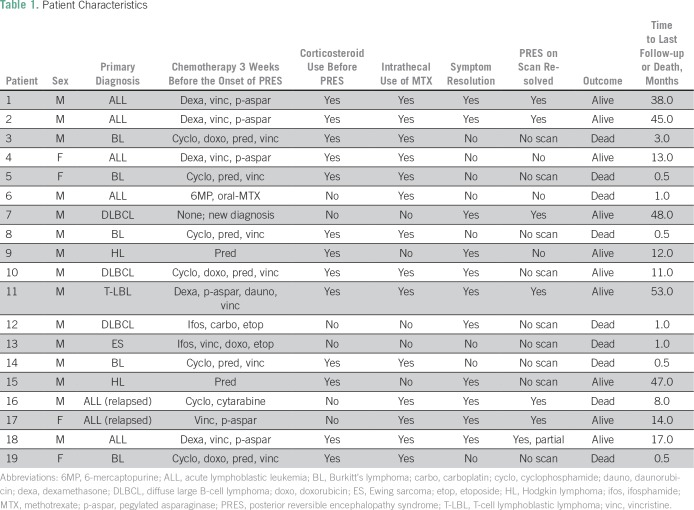
Patient Characteristics

**Table 2:**
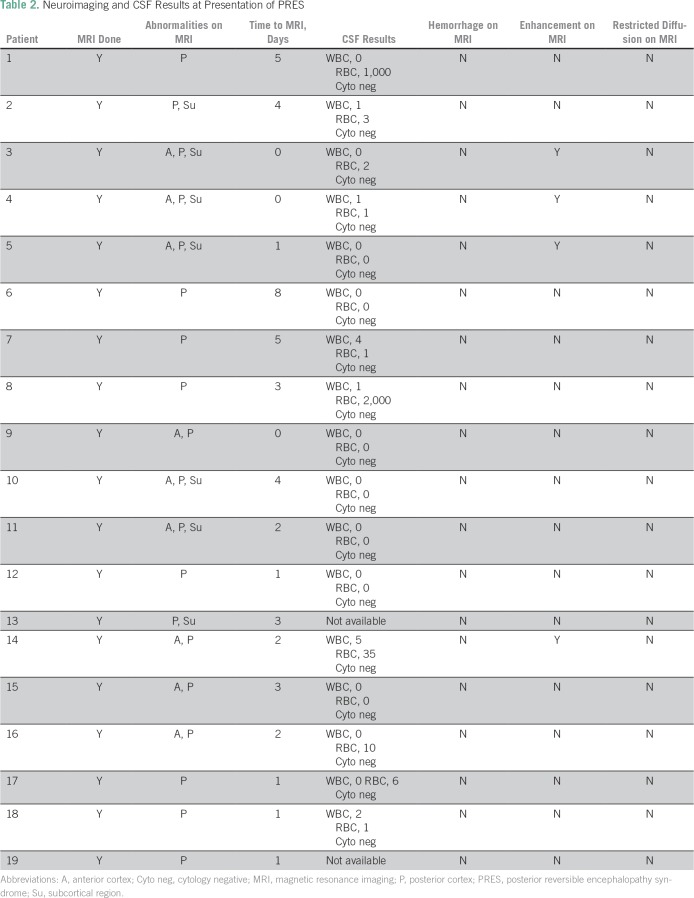
Neuroimaging and CSF Results at Presentation of PRES

During January 2013 to June 2016, 1,208 children were diagnosed with cancer at our single institution. On average, approximately 400 new patients present per year. Our institution is the only freestanding cancer center in the country. Patient referrals are made not only within the country but also from neighboring Afghanistan. PRES was seen in 2% of children with lymphoma (n = 545), 1.6% with leukemia (n = 306), and 0.28% with solid tumors (n = 357) with the exclusion of brain tumors. The difference in incidence rates was significant only for lymphomas versus solid tumors (*P* = .018).

Seizures and hypertension were the most common symptoms seen in > 90% of the patients (Table 3). Altered mental status was seen in 12 patients, visual disturbances in five, and cortical blindness in two. Types of seizures varied. EEG could only be performed in six patients, whereas the rest were not done because those children were clinically unwell to go to the EEG facility. Sixteen patients were started on antiepileptics that included phenytoin, levetiracetam, or both. Initially, no consensus existed on first-line antiepileptic therapy, hence the variation among patients in our center. We now use levetiracetam as the medication of choice. For refractory seizures, phenytoin is added after consultation with the neurologist. Sixteen of the 19 patients received intrathecal chemotherapy. Seven were still receiving antiepileptic medications at the time of this review. Two patients were successfully weaned after 6 months of treatment.

**Table 3:**
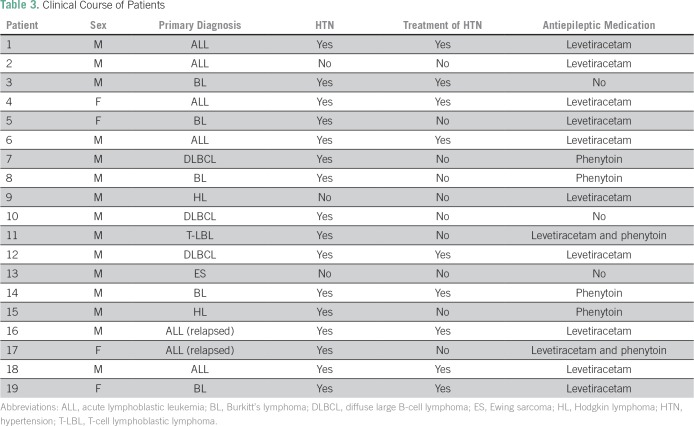
Clinical Course of Patients

Symptoms of PRES resolved in 11 of the 19 patients. Eight did not experience resolution of symptoms, seven of whom died, and one (patient 4) has quadriplegia with no vision or speech and hydrocephalus that requires a ventriculoperitoneal shunt. All these patients were very ill and receiving ventilatory support. PRES was not the immediate cause of mortality. As a result of the management of PRES, most of the chemotherapeutic regimens were paused while patients were imaged, treated, and stabilized. Patients 3, 5, and 8 died as a result of multiorgan failure. Patients 6, 13, 14, and 16 had respiratory failure that led to death. Patients 12 and 19 died as a result of cardiac arrest. Ten patients who are alive are doing well clinically except for patient 4. These patients have returned to school with no active PRES-related complaints.

Our standard MRI protocol includes T2, FLAIR, gradient recalled echo, diffusion weighted imaging, and T1 pre- and postcontrast imaging. T2/FLAIR high signal and T1 low to isointensive signal abnormalities were noted with or without additional findings. High signal change was noted on diffuse weighted imaging, but no corresponding low signal was seen on apparent diffusion coefficient. No high T1 or low gradient recalled echo was found to suggest hemorrhage.

**Fig 1:**
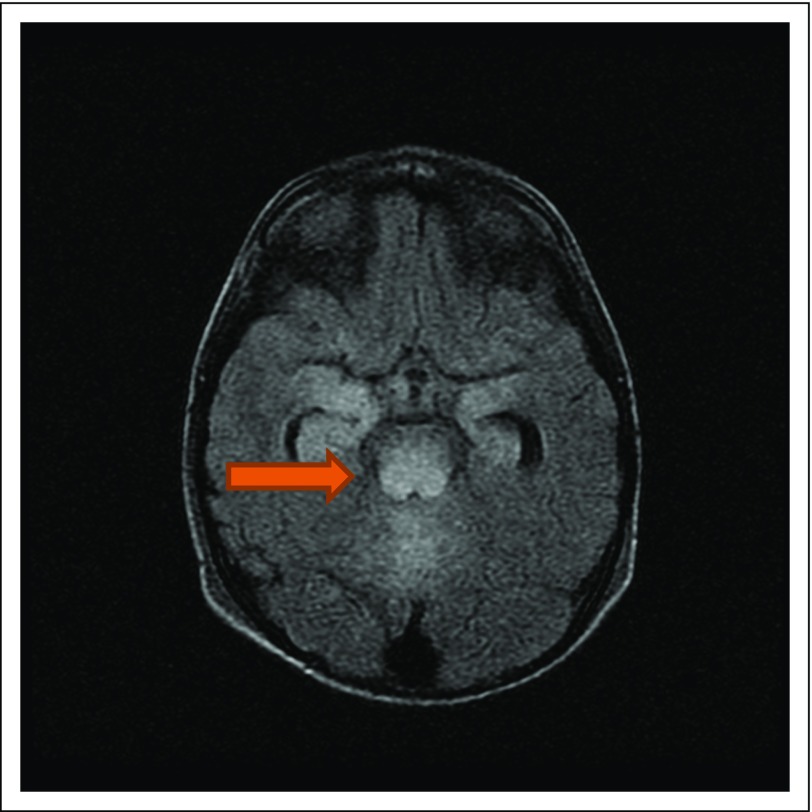
Bilateral symmetrical subcortical white matter involvement of the occipital and parietal lobes without diffusion restriction or hemorrhage.

**Fig 2:**
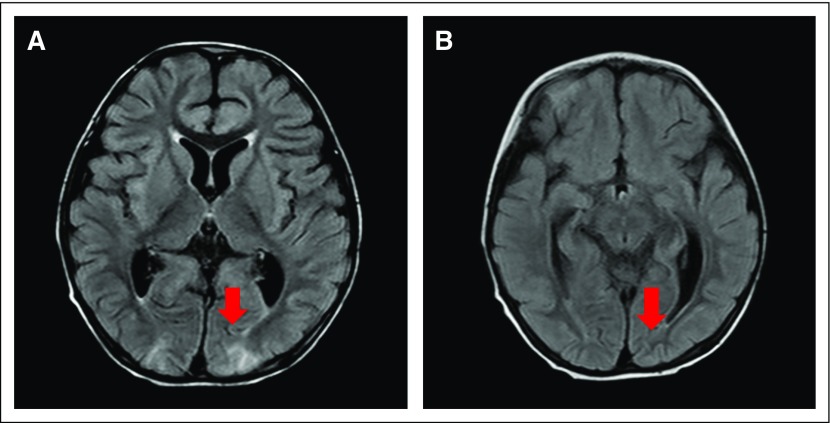
Bilateral occipital lobe involvement (A and B), typical area of posterior reversible encephalopathy syndrome involvement.

**Fig 3:**
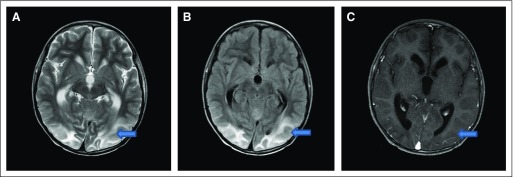
(A, B, and C) Multifocal involvement with combination of occipitocerebellar, occipitoparietotemporal, and fronto-occipitotemporal lobes or isolated involvement of the parietal and temporal lobes.

The most common finding was bilateral symmetrical subcortical white matter involvement of the occipital and parietal lobes without diffusion restriction or hemorrhage (Fig 1). This distribution of involvement typical of PRES was seen in seven patients (37%). The second most common pattern was bilateral occipital lobe involvement, again typical area of PRES involvement (Fig 2), seen in five patients (26%). Less common patterns were either a multifocal involvement with combination of occipitocerebellar, occipitoparietotemporal, and fronto-occipitotemporal lobes or isolated involvement of the parietal and temporal lobes (Fig 3). No patients had central PRES given that no abnormalities were seen in the brainstem or basal ganglia. Eight scans showed resolution of findings on reimaging. We prefer reevaluation scans within 4 to 6 weeks of diagnosis. We have been unable to set up a definitive follow-up scan time after initial diagnosis.

## DISCUSSION

Pediatric patients with cancer are at risk for PRES, especially those with hematologic malignancies.^6,10-13^ This study shows that pediatric patients with solid tumors are also at risk. Common symptoms include seizures, hypertension, and altered mental status.^1^ All children with infection and electrolytes imbalance were assessed for CNS involvement. With better clinical awareness and MRI availability at our institution, we are recognizing more cases of PRES. Most of our patients developed PRES during induction chemotherapy. The diagnosis could not be related to a certain type of chemotherapeutic agent because a multitude of agents had been used for various diagnoses.^10,14^ Nonetheless, these toxic medications could have led to endothelial damage that resulted in PRES.

Twelve patients had a history of intrathecal chemotherapy given within the last 3 weeks of developing PRES. The reason for this timing is unclear. Direct endothelial dysfunction with a subsequent breach of the blood-brain barrier is also a proposed mechanism of PRES and can occur with induction systemic and intrathecal chemotherapy. The most common systemic chemotherapy our patients received before diagnosis of PRES was intrathecal methotrexate, dexamethasone, cyclophosphamide, and pegylated asparaginase. Of note, these agents were reintroduced after resolution of PRES with no recurrence of symptoms.

Hypertension is believed to be one of the key factors in PRES.^5,6^ Increase in blood pressure and disruption of the blood-brain barrier, which leads to vasogenic edema, are hypothetical causes of PRES.^15^ Most pediatric patients treated for cancer receive corticosteroids. Corticosteroid-induced hypertension seems to play a role in the development of PRES.^16^ Additional causes of hypertension that lead to PRES are renal dysfunction as a result of either direct renal involvement (as seen in two of our patients with Burkitt’s lymphoma) or secondary to tumor lysis given the disease burden. None of our patients with leukemia had high enough WBC counts to cause hyperviscosity syndrome that might have led to PRES.

The current results suggest that blood pressure should be monitored carefully to avoid corticosteroid-induced hypertension that might lead to PRES. All patients with suspected PRES should be evaluated with MRI, including FLAIR and T2-weighted imaging.^17,18^ Follow-up imaging is necessary along with reversibility of neurologic deficits.^6,19^ In the current cohort, we could obtain follow-up MRIs in 10 patients. Most who had symptom resolution also had scans that showed improvement (patient 9 improved after 8 weeks); two had interval progression.^20,21^ One of the two patients with interval progression died, and the other (patient 4) was discharged after a prolonged intensive care unit (ICU) admission. The nine patients who did not undergo follow-up imaging died as a result of medical complications related to their primary disease.

Our incidence of PRES at 1.6% in children with leukemia and 0.28% in those with solid tumors other than brain malignancies were similar to that reported by Khan et al.^22^ Our highest incidence of PRES (2%) was in patients with lymphoma. These children are cachectic on presentation, with most needing an ICU admission, dialysis, and therapeutic rasburicase. In our developing world, good primary health care is scarce. Most of the physicians outside our center tend to perform either open biopsies or operations before appropriate work-up. After pathology confirms the diagnosis is when patients are referred to us. This observation has led us to believe that PRES is more of an issue in very sick patients secondary to endothelial damage as a result of inflammatory mediators and cytokines. Prospective studies are needed to better elucidate the role of these observations about PRES. Our center also currently does not have a pediatric ICU. Adult-trained intensivists manage these sick children. A pediatric ICU is important for improving outcomes in critically ill oncology patients.

PRES was seen in approximately three times as many male patients as female patients. This sex ratio can be explained by the gender inequality practices of our society. The gender gap in poor countries like ours favors the male child with respect to education, health, and freedom. In their series of patients with kidney disease and PRES, Gera et al^23^ showed a similar male pattern. In patriarchal society like ours, the desire to have sons is greater, with more investment in their health and well-being. We see more male children brought to tertiary care facilities for treatment. The monetary return for saving a girl’s life is not enough to invest in her medical treatment, which is abandoned by most families.

A number of retrospective reviews on PRES exist in the pediatric oncology literature. Kim et al^24^ showed that PRES mostly occurs in patients who undergo induction chemotherapy for acute leukemia (47.4%). Morris et al^6^ showed that other malignant diagnoses, such as those we studied, also can be complicated by PRES. In our cohort, 21% of patients with leukemia (n = 4), 36.8% with non-Hodgkin lymphoma (n = 7), and 5.3% with Hodgkin lymphoma (n = 1) who received induction chemotherapy developed PRES (n = 12 [63.1%]). Our series has reported young and sick patients at the highest risk for developing PRES during initiation of chemotherapy.^20,23^

Conclusive data is lacking on the best treatment approach for PRES. Studies have described symptom-directed therapy that includes antihypertensive and antiseizure medications.^6,13^ Patients were continued on antiepileptics for approximately 6 months after being seizure free. Antihypertensive medications for symptom control were used over a shorter period. None of our patients had long-term clinical sequelae.^10,25^

To conclude, we have a low threshold in suspecting PRES as a complication in children treated for cancer. Our clinical practice is to obtain a lumbar puncture, laboratory tests, and an MRI and to administer antiepileptic medication, preferably levetiracetam, after the first seizure in patients with suspected PRES. PRES generally is a reversible condition, but in patients who present with advanced stages of cancer, we need additional study about its contribution to mortality, if any. The neurologic outcomes of patients with PRES during cancer treatment also should be studied prospectively.
